# Nanophotonic perfect absorber with ultra-broadband terahertz-to-infrared response via hybrid-material design for advanced optical sensing

**DOI:** 10.1371/journal.pone.0342168

**Published:** 2026-02-06

**Authors:** Musa N. Hamza, Mohammad Tariqul Islam, Sunil Lavadiya, Iftikhar ud Din, Bruno Sanches, Slawomir Koziel, Syeda Iffat Naqvi, Ali Farmani, Abinash Panda, Md. Shabiul Islam

**Affiliations:** 1 Department of Physics, College of Science, University of Raparin, Sulaymaniyah, Iraq; 2 Department of Electrical, Electronic and Systems Engineering, Faculty of Engineering and Built Environment, Universiti Kebangsaan Malaysia (UKM), Bangi, Selangor, Malaysia; 3 Computer and Information Sciences Research Center (CISRC), Imam Mohammad Ibn Saud Islamic University (IMSIU), Riyadh, Saudia Arabia; 4 Department of Information and Communication Technology, Marwadi University, Rajkot, Gujarat, India; 5 Telecommunication Engineering Department, University of Engineering and Technology, Mardan, Pakistan; 6 Department of Electronic Systems Engineering, Escola Politécnica da Universidade de São Paulo, São Paulo, Brazil; 7 Engineering Optimization & Modeling Center, Reykjavik University, Reykjavik, Iceland; 8 Faculty of Electronics, Telecommunications and Informatics, Gdansk University of Technology, Gdansk, Poland; 9 Department of Telecommunication Engineering, University of Engineering & Technology Taxila, Taxila, Pakistan; 10 Department of Electronics Engineering, Lorestan University, Khorramabad, Iran; 11 Department of Electronics and Communication Engineering, CMR Institute of Technology, Bengaluru, India; 12 Centre for Advanced Devices and Systems, Centre of Excellence for Robotics and Sensing Technologies, Multimedia University, Cyberjaya, Selangor, Malaysia; 13 Faculty of Artificial Intelligence and Engineering, Multimedia University, Cyberjaya, Selangor, Malaysia; Purdue University, UNITED STATES OF AMERICA

## Abstract

The terahertz (THz) frequency range has gained significant attention in recent years, particularly for applications in biological diagnostics, remote sensing, security systems, and wireless communications. One key advantage of THz radiation is that it is safer than X-rays while offering higher data rates and enhanced channel capacity. THz systems encapsulate several components, including absorbers, which play a crucial role in stealth technologies, detection, and high-resolution imaging. Many absorber designs in the literature are based on metamaterials; however, these structures tend to be physically large and thick, limiting their integration into devices. This research introduces an innovative, compact THz-range sensor designed for biomedical applications. The sensor features a geometrically simple structure, utilizing silver (Ag) and nickel (Ni) resonators embedded on a silicon dioxide (SiO2) dielectric substrate. The device measures only 100 × 100 nm², with the Ag, SiO2, and Ni layers totaling just 26 nm thickness. This material and geometric arrangement achieve near-perfect absorptivity (>99.9%) across the operating range up to 30 THz. Extensive numerical studies demonstrate the sensor’s excellent performance, analyzed through surface current, electric, and magnetic field distributions. Compared to state-of-the-art benchmarks, comprehensive comparative studies reveal the sensor’s superior performance in terms of operating range, compact size, absorption efficiency, and angular stability. Its exceptional sensitivity and ability to detect subtle changes in tissue refractive index make it ideal for early-stage cancer detection and other biomedical applications. Additionally, it is well-suited for real-time detection of environmental pollutants and security screening.

## 1. Introduction

The THz frequency range, situated between electronics and photonics, offers unique advantages due to its positioning. It covers wavelengths from 3 mm to 10 μm, and its wide bandwidth (around 30 THz) enables it to be useful in a variety of fields such as in astronomy for space exploration, as THz radiation can penetrate cosmic dust and other interstellar materials [1]. Also, THz radiation, being less harmful than X-rays, is valuable for safe imaging techniques, making it applicable in both biological diagnostics, sensing [2–13] and security systems. Moreover, higher carrier frequencies in the THz band allow for increased data rates and enhanced channel capacity, especially for short-range and ultra-wideband communications, supporting future advancements in wireless communication technologies [14]. So several possible applications lead to a promising research in this frequency band in recent years.

An integrated THz diagnostic platform fundamentally depends on the synergistic performance of its constituent components, among which electromagnetic absorbers serve as a critical element. These structures are engineered to efficiently attenuate incident electromagnetic waves over specific spectral bands and are indispensable for applications such as non-invasive biomedical imaging, biomolecular sensing, stealth technologies, and spectral fingerprinting of pathological tissues [15]. Over the past decade, extensive research efforts have led to the development of numerous absorber configurations spanning frequency ranges from gigahertz (GHz) and terahertz (THz) to lower petahertz (PHz) regimes, particularly for early cancer diagnostics, where the detection of subtle dielectric perturbations caused by malignant cells is paramount [16–61].

However, the vast majority of these absorbers are based on microscale resonant metamaterials, multilayered dielectric-metal stacks, or classical plasmonic designs, which—despite their functional merit—often suffer from limitations such as increased thickness, limited spectral tunability, and restricted spatial resolution. These constraints inhibit their practical deployment in next-generation lab-on-chip platforms and portable biomedical devices. In contrast, nanophotonic absorbers provide a transformative solution by enabling deep-subwavelength light confinement, high field enhancement, and ultrathin integration, thereby dramatically enhancing the sensor’s spectral selectivity, sensitivity to refractive index changes, and compactness.

Nano-photonics absorbers in the terahertz (THz) region are advanced materials designed to absorb terahertz waves effectively. In particular, the integration of nano-photonics with THz technologies allows for precise control over light-matter interactions at the nanoscale, enabling the development of highly efficient absorbers. Mainly nano-photonic THz absorbers rely on resonance effects, where incident THz waves match the natural frequencies of the structures. This matching enhances the interaction between the electromagnetic wave and the material, leading to efficient absorption. Thus, their ability to manipulate and absorb THz radiation at the nanoscale makes them crucial in developing next-generation systems for imaging, communication, and sensing [62,63]. Nanophotonic absorbers in the THz range typically operate on the principle of resonant absorption, where the nanostructures are tuned to resonate at specific THz frequencies, resulting in efficient absorption of energy. Many THz absorbers use layered designs where each layer serves to reflect or absorb specific frequencies. These can be optimized to achieve broadband absorption over a wide range of THz frequencies [64,65].

Recent research has focused on developing highly efficient and broadband absorbers, largely driven by the growing demand for advanced sensing, imaging, and communication systems operating in the THz spectrum [65]. The investigation of materials with suitable conductivity and permittivity in the THz frequency range is crucial for advancing terahertz technology. The ongoing research into new materials and their nanoscale structuring is driving progress in THz absorbers. Most of the early absorbers were made of metal metamaterials. Conventional noble metals, such as gold and silver are extensively investigated alongside silica [66]. Also, some research works explored two-dimensional (2D) materials such as phosphorene, borophene, and silicene [67–69] for potential usage in THz frequencies. Narrow-band and broadband absorbers both play essential roles in various advanced technologies, each catering to different applications based on their absorption capabilities in the terahertz (THz) range. Narrow-band absorbers are designed to absorb electromagnetic waves efficiently at specific frequencies or within a narrow spectral range primarily aiming bio detection, mineral exploration, artificial intelligence, and hyperspectral imaging [70–72], whereas, broadband absorbers are engineered to absorb a wide range of frequencies, making them suitable for applications where efficiency across a broad spectrum is required. The ability to absorb over a wide band makes these absorbers crucial for sensing, radiation emission, cloaking devices and photovoltaic batteries [73–78]. Various geometries and several materials have been investigated to form THz wave absorbers as discussed above. However, these works demonstrated complex microscale structures mostly designed on the basis of metamaterials. The thick and large sizes of structures would impede device integration. Therefore, studies on absorbers that demonstrate simple nanoscle structures, high absorption rates, and a broad relative bandwidth are still needed for further investigation of the practical applications.

This study presents an ultra-compact nanophotonic perfect-absorber biosensor engineered to operate across an exceptionally broad spectral window spanning 100 GHz to 30 THz. The proposed design, with a footprint of only 100 × 100 nm², represents a significant advancement in nanoscale terahertz device engineering by demonstrating near-unity absorption over the entire band. Such performance is achieved through the strategic integration of nickel and silver resonant layers on a silicon dioxide substrate, enabling precise control of electric and magnetic field confinement at deep-subwavelength scales.

The sensor’s nanophotonic architecture exhibits strong sensitivity to slight variations in the refractive index of biological tissues, making it highly suitable for biomedical diagnostics, including the early detection of malignancies where refractive-index contrast serves as a key physiological indicator. Beyond biomedical applications, the device’s broadband operation and near-perfect absorption open pathways for multifunctional use in environmental sensing—where identifying trace pollutants or atmospheric chemicals demands both high sensitivity and wide spectral coverage—and in security and defense systems requiring reliable detection of concealed objects or hazardous materials.

By simultaneously achieving extreme miniaturization, ultrabroadband response, and robust spectral stability, the proposed structure addresses long-standing limitations in THz and mid-infrared absorber technologies. The sensor therefore constitutes a technically significant contribution to the field, offering a versatile, high-performance platform capable of supporting next-generation nanophotonic, biomedical, and spectroscopic systems.

## 2. Geometrical architecture and alignment of the incident electromagnetic excitation

**Fig 1:**
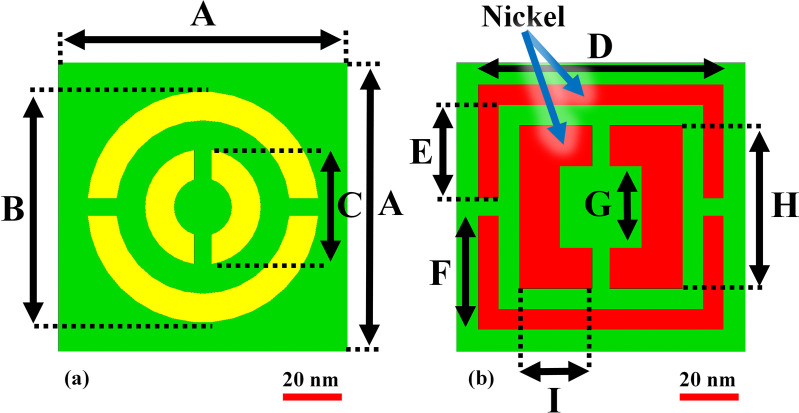
Schematic of the proposed nano-photonics perfect absorber: (a) front view depicting silver resonators, (b) rear view illustrating the nickel ground layer.

Proposed structural configuration displays in Fig 1. Terahertz biosensor, which is fabricated of nickel/silver/SiO_2_ which can be used in terahertz region. Here, THz radiation is coupled to the structure and to benchmark of the structure, absorption spectrum is calculated. As we expect the perfect absorption is met in DC frequency and as frequency tends to higher value the absorption has an exponential behavior in turn leads to zero value. The center of the structure has strong THz-mater interaction and has main role in this structure.

The incident electromagnetic field refers to the initial electromagnetic field that interacts with a medium or object. Its orientation is determined by the direction of the electric and magnetic fields that make up the wave. In general, the orientation of an electromagnetic wave is perpendicular to the direction of wave propagation, with the electric and magnetic fields oscillating in planes that are perpendicular to each other. This orientation is crucial in understanding how the electromagnetic field interacts with materials.

**Fig 2:**
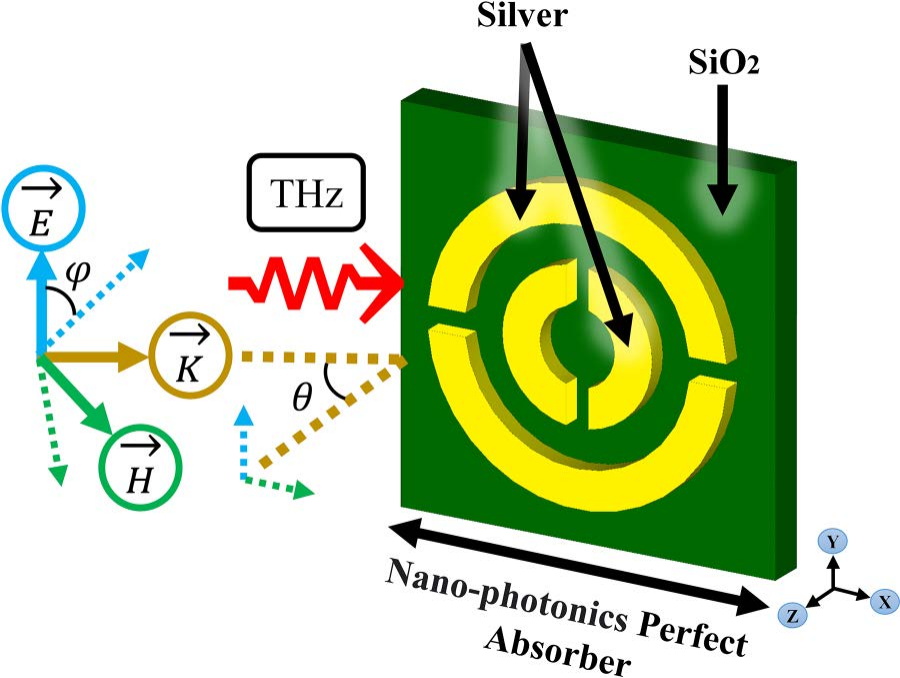
Schematic illustration of the proposed geometry and excitation conditions of the incident electromagnetic field used to achieve enhanced absorption performance.

As we know, the angle of incident THz field has crucial role to gain maximum absorption with high sensitivity, Fig 2 provides nano-photonics perfect absorber. As can be seen, THz ray easily trapped in the surface of the structure and the insertion loss tends to zero value and this is a novelty of structure.

In Table 1 geometrical parameters of the proposed nano-photonics perfect absorber is provided. The footprint of the proposed structure is 100 × 100 nm^2^. With such a structure, fabrication process from experimental point of view can be easily done. Also, the thickness of Ag, SiO2 and Ni are 26 nm, which can be done by fabrication techniques.

**Table 1:** Detailed set of tunable geometrical and material parameters used for optimizing the proposed nanophotonic absorber design.

Parameter	Value (nm)	Parameter	Value (nm)
**A**	100	G	28.3
**B**	80	H	56.6
**C**	40	I	25.3
**D**	85	SiO2 Thick	14
**E**	32.3	Ag Thick	6
**F**	39.4	Ni Thick	6

## 3. Absorption properties and analysis

In this study, two distinct nanophotonic absorber configurations—Model 1 and Model 2—were intentionally selected to investigate how controlled geometric variations influence the broadband absorption characteristics of ultra-thin THz-to-infrared metamaterial structures. Both models were designed to operate within the same physical footprint and material constraints, enabling a systematic evaluation of their electromagnetic behavior across a wide spectral range (0–30 THz).

**Fig 3:**
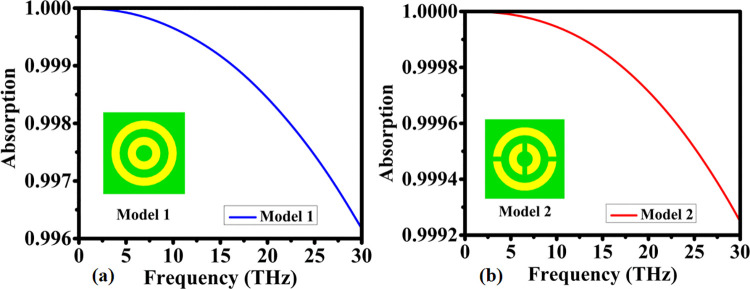
Comparison of the absorption responses obtained from two different structural configurations: (a) absorptive behavior of Configuration I and (b) absorptive behavior of Configuration II.

Model 1, consisting of two concentric circular resonators, was chosen as a geometrically symmetric baseline structure. Circular geometries are widely recognized in nanophotonics for their ability to support uniform surface current distributions, stable dipole and higher-order resonance modes, and inherent polarization insensitivity. These characteristics make concentric rings an ideal starting point for establishing whether a strongly confined electric and magnetic field response can be obtained in an ultrathin absorber. Consistent with these theoretical expectations, Model 1 achieves exceptional absorptivity, exceeding 99.9% in the 0–15 THz range and maintaining absorption above 99.6% up to 30 THz. The smooth resonance progression observed in Fig 3(a) confirms that symmetry-driven impedance matching plays a central role in enabling robust broadband performance.

Model 2 was introduced as a purposeful geometric evolution of Model 1. By incorporating two opposing cuts in each ring, Model 2 breaks the rotational symmetry present in Model 1, thereby enabling additional pathways for field localization and improving the interaction between the incident electromagnetic wave and the resonant structure. This controlled asymmetry enhances electromagnetic field confinement and facilitates more efficient impedance matching across the broadband spectrum, which is essential for achieving uniform and stable absorption in ultrathin nanophotonic devices. As demonstrated in Fig 3(b), Model 2 maintains extremely high absorption efficiency—over 99.98% below 15 THz and above 99.92% across the higher 15–30 THz band. These results confirm that subtle geometric perturbations can fine-tune the absorber’s response without compromising its compact size or structural simplicity.

Comparing the two models highlights the interplay between geometric symmetry, resonance hybridization, and spectral broadening. Model 1 provides a canonical symmetric design with smooth, wideband absorption, making it suitable as a baseline for studying the influence of symmetry. Model 2, in contrast, demonstrates how targeted structural modifications can enhance the density of electromagnetic modes and optimize field confinement, resulting in slightly improved absorption uniformity at higher frequencies. The choice between these geometries ultimately depends on the intended biosensing application and the required sensitivity across specific spectral regions.

Overall, the inclusion of both models enables a rigorous understanding of how nanoscale geometric engineering affects absorption behavior, and it provides the scientific foundation upon which the final optimized nanophotonic absorber was developed.

**Fig 4:**
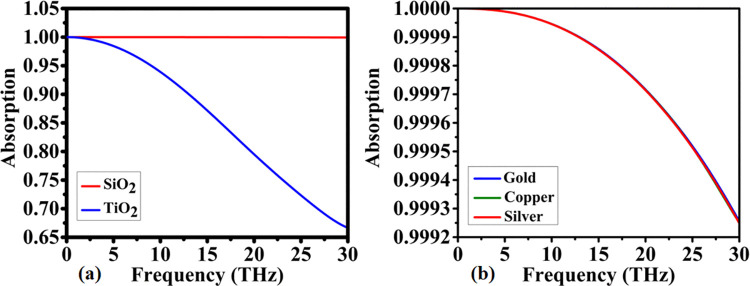
Influence of material variation on the absorption characteristics of the designed structure, showing (a) substrate-dependent absorption spectra and (b) resonator-material-dependent absorption spectra.

The results presented in Fig 4(a) provide a comparative evaluation of the absorptive responses obtained using SiO₂ and TiO₂ substrates, highlighting their influence on broadband absorption efficiency over the considered frequency spectrum. SiO2 demonstrates outstanding absorption, maintaining 100% efficiency across the entire 0–30 THz range. This broad and effective coverage ensures that a significant portion of incoming energy is fully absorbed, making SiO2 an ideal substrate for applications requiring wide-spectrum absorption. In contrast, TiO2 shows a weaker absorption response. From 0 to 10 THz, its absorption remains above 95%, though it falls short of SiO2’s performance. Between 10 and 20 THz, TiO2 experiences a notable decrease, with absorption dropping to just above 80%. Beyond 20 THz, the absorption continues to decline, reaching 60% at 30 THz. Overall, TiO2’s absorption performance is lower across the entire frequency spectrum compared to SiO2. This analysis emphasizes SiO2 as the superior substrate material for broad-spectrum absorption, providing consistently high efficiency. While TiO2 shows reasonable absorption at lower frequencies, its performance remains significantly inferior to SiO2 in terms of wide-spectrum absorption.

The decrease in absorption between SiO2 and TiO2 can be attributed to their differences in refractive index and dielectric properties, key factors in nanophotonics. SiO2 has a lower refractive index and dielectric constant, which allows for better electromagnetic wave coupling and absorption. In contrast, TiO2’s higher refractive index leads to greater reflectivity, causing lower absorption at higher frequencies. The material’s dielectric loss also influences its ability to absorb energy. TiO2’s higher dispersion at increased frequencies results in less effective electromagnetic wave interaction, which explains the significant drop in absorption beyond 10 THz.

The analysis of absorption responses in resonating materials such as gold, copper, and silver, as shown in Fig 4(b), reveals significant absorption efficiency across a broad frequency range. These materials exhibit high absorption rates, with absorption exceeding 99.99% in the 0–10 THz range. Beyond this range, absorption decreases slightly, maintaining approximately 99.97% efficiency from 10 to 20 THz, and further decreasing to about 99.92% between 20 and 30 THz. Despite these reductions, the absorption spectrum remains relatively consistent over a wide frequency band. These variations in absorption performance are attributed to the unique electronic structures and plasmonic properties of gold, copper, and silver. As noble metals, gold and silver possess high electrical conductivity and reflectivity, which enhance their interaction with electromagnetic waves. The efficient absorption over a wide frequency spectrum is primarily driven by surface plasmon resonance (SPR), where conduction electrons on the metal surface resonate with incident light, facilitating energy absorption. Silver, in particular, is renowned for its superior plasmonic properties, especially in the visible spectrum, making it a preferred material for applications within this frequency range.

## 4. Nano-photonics structure design and parameter analysis

This section summarizes the nano-photonics structure design, detailing simulations on absorption characteristics, resonator and substrate thickness, sensor dimensions, and material properties (permeability and permittivity). The results, presented via graphical analysis, highlight key factors affecting absorption efficiency and overall performance of the proposed absorber.

### 4.1. Absorption spectra and parameter simulation

**Fig 5:**
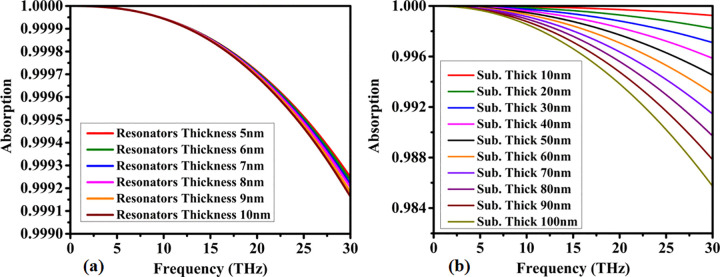
Parametric investigation of thickness effects on absorptive performance, illustrating (a) resonator layer thickness variation and (b) substrate layer thickness variation.

Different parametric analyses help identify the optimum response of a structure. The absorption analysis for varying resonator thickness is shown in Fig 5(a). The analysis is carried out for a thickness range of 5 nm to 10 nm. The absorption response remains consistent across all variations. Overall, more than 99% absorption is achieved throughout the entire region.

The substrate thickness variation response is shown in Fig 5(b). The substrate thickness is varied from 10 nm to 100 nm, with a step size of 10 nm. The absorption response shows that as the thickness increases, the absorption decreases. A thickness of 10 nm provides maximum absorption, while 100 nm results in minimum absorption.

**Fig 6:**
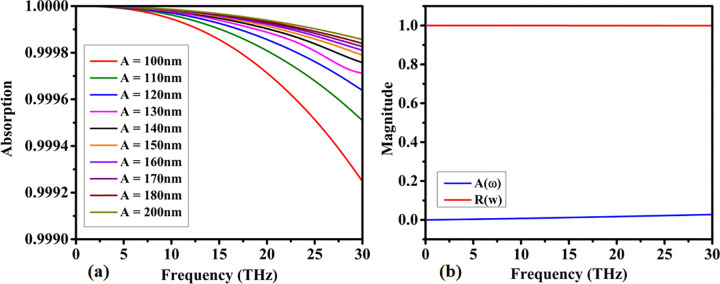
Influence of geometrical scaling on absorptive behavior: (a) effect of sensor dimensional variations on the absorption response, and (b) corresponding reflection and absorption spectra of the proposed absorber.

The overall structure size plays an important role in the absorption response. The absorption response for the variation of parameter A is shown in Fig 6(a). Parameter A varies from 100 nm to 200 nm. The absorption response is not significantly affected by this variation. Overall, more than 99% absorption is achieved for all variations of A.

The absorption and reflectance response of the proposed structure is shown in Fig 6(b). It is observed that they are complementary to each other. Peak absorption of 1 and near-zero reflectance are achieved.

### 4.2. Analysis of permeability, permittivity, and simulated responses

**Fig 7:**
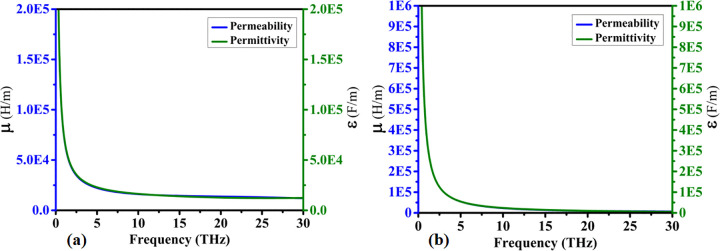
Extracted effective electromagnetic properties of the designed absorber, showing (a) real parts of the permeability (μ) and permittivity (ε), and (b) imaginary parts of the permeability (μ) and permittivity (ε).

The nanophotonics properties based on permeability and permittivity are shown in Fig 7. The real and imaginary responses represent how the permeability and permittivity values change concerning frequency. High values of μ and ε are achieved at lower frequencies, while lower values of μ and ε occur at higher frequencies.

The maximum real value of 2.0E5 and the minimum real value of 1.5E5 are achieved, as shown in Fig 7(a). The maximum imaginary value of 1E6 and a minimum value close to zero are shown in Fig 7(b).

**Fig 8:**
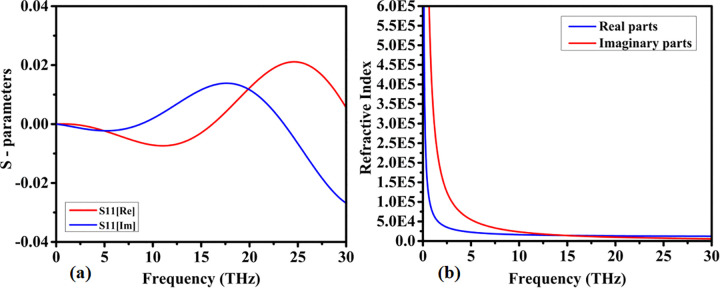
Numerically obtained electromagnetic parameter characteristics of the proposed structure: (a) real and imaginary components of the reflection coefficient (S₁₁), and (b) real and imaginary components of the effective refractive index.

*/S*_11_/ is crucial in determining how well an absorber decreases reflections. Better impedance matching represents decreased reflection and enhances absorption. The imaginary component, which represents the phase shift of the reflected wave, exposes the absorber’s reactive surface (capacitive or inductive). For an effective absorber, the real and imaginary components of S11 should be lowered such that more incoming energy is absorbed rather than reflected. The real and imaginary response of the reflection coefficient is shown in Fig. 8(a). The peak real value of 0.01 was observed at 18THz and 0.02 of the imaginary value was achieved at 25.5THz.

The refractive index is a complex number with real and imaginary components that are important. The real component of the refractive index determines the wave’s passage through the absorber material, slowing it down. The imaginary component of the refractive index indicates the amount of wave energy that the material absorbs as heat. An effective absorber has a higher imaginary part (as shown in Fig 8(b)), which absorbs more energy and reduces reflections. These components assist the absorber reduce reflections and properly distribute incoming wave energy. A peak value of 6.0E5 is achieved for lower frequency, the response reduces for higher frequency.

**Fig 9:**
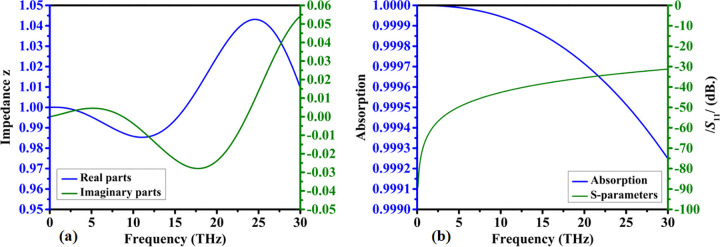
Simulated impedance and scattering behavior of the proposed design, illustrating (a) normalized impedance response (z) and (b) reflection coefficient magnitude (|S₁₁|) expressed in decibels.

The real and imaginary components of impedance are important. The real value of impedance determines how much EM wave energy is absorbed and converted into heat. The phase shift of EM fields is controlled by the absorber’s reactive component. The imaginary component should be chosen to balance capacitive and inductive effects such that the absorber dissipates energy across a wide frequency range. Fig 9(a) shows the 1.04 Peak real value of impedance achieved at 25THz and the imaginary value is increased for higher frequency. The absorption and reflection coefficient response is shown in Fig 9(b). Peak absorption is shown for lower frequency and the reflection coefficient reduces as frequency increases.

## 5. Electromagnetic field distribution and surface current characteristics of the nanophotonic absorber

This section examines the spatial distributions of electromagnetic fields and surface current densities within the proposed nanophotonic absorber, offering insight into the underlying absorption mechanisms. Color-mapped simulations are employed to visualize the real and imaginary components of both electric and magnetic fields, highlighting their interaction with the metallic resonators and the nickel-based ground plane across multiple observation planes.

### 5.1. Visualization of electromagnetic field distributions

**Fig 10:**
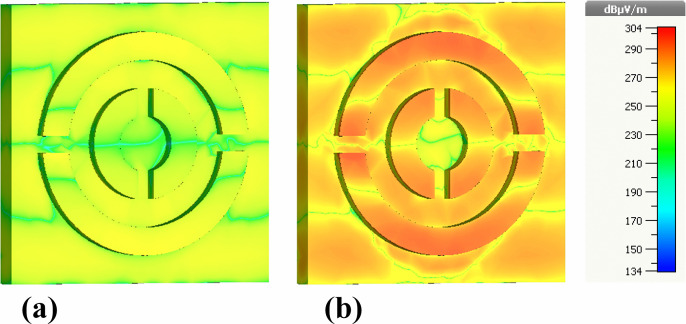
Front-plane visualization of the silver-based resonator array illustrating nanophotonic electric-field distributions: (a) real component of the out-of-plane electric field (Ez) and (b) imaginary component of the out-of-plane electric field (Ez).

**Fig 11:**
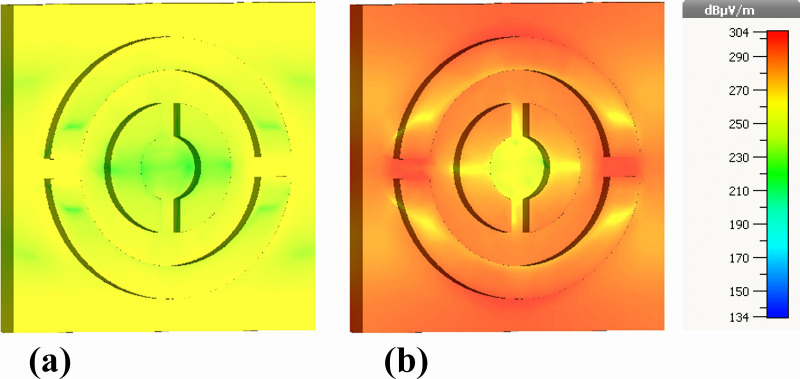
Color-mapped front-view representation of the magnitude of the electric field within the silver resonators, showing (a) real part of |E| and (b) imaginary part of |E|.

**Fig 12:**
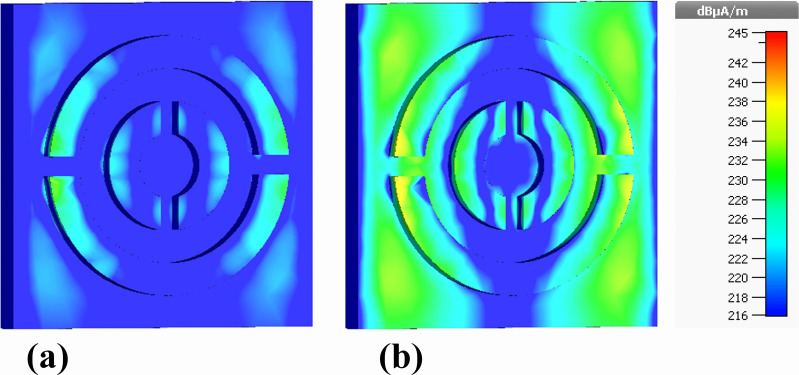
Front-view nanophotonic field maps depicting magnetic-field behavior in the silver resonator structure: (a) real component of the out-of-plane magnetic field (Hz) and (b) imaginary component of the out-of-plane magnetic field (Hz).

**Fig 13:**
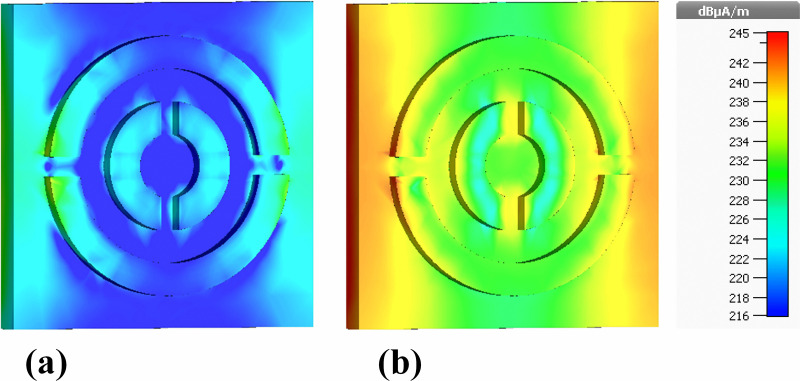
Spatial distributions of the magnetic-field magnitude across the silver-based resonator array obtained from front-view simulations, illustrating (a) real part of |H| and (b) imaginary part of |H|.

Figs 10–13 present the numerically simulated electric- and magnetic-field distributions established within the silver resonator array constituting the top layer of the proposed ultra-broadband biosensor, which operates over the 0.1–30 THz frequency range. The field profiles demonstrate pronounced electromagnetic confinement within the concentric split-ring resonators, indicating the excitation of localized plasmonic modes. These modes play a central role in achieving near-perfect absorption and contribute directly to the enhanced sensitivity of the biosensing platform.

Fig 10 shows the real and imaginary components of the longitudinal electric field (Ez). Both maps share a common intensity scale from 134 to 304 dB μV/m. Weak perturbation of the incident wave appears in blue–green tones across the outer substrate regions, while progressively stronger confinement emerges near the metal–dielectric interfaces of the resonant rings. The field intensifies from green to yellow and finally to orange and red at the inner ring edges and split gaps, representing the principal hot spots where plasmonic charge accumulation is strongest.

The real component (Fig 10(a)) exhibits more symmetric transitions around the resonators, whereas the imaginary component (Fig 10(b)) displays broader high-intensity red zones, indicating enhanced reactive energy storage and more pronounced oscillatory Plasmon behavior. Together, these features confirm the existence of tightly confined Ez-modes essential for ultrahigh absorption and strong analyte–field interaction.

Fig 11 presents the magnitude of the electric field (|E|), again separating real and imaginary components with the same 134–304 dB μV/m range. Both maps show moderate field levels (yellow–green) in the background plane and strong intensification toward the inner circular boundaries and narrow gap regions. These zones exhibit bright yellow to deep red tones, indicating |E|-field hot spots approaching ~304 dB μV/m.

The imaginary component reveals additional high-reactance regions, signifying enhanced oscillatory energy exchange between the resonator and the incident wave. These field patterns resemble those in Fig 10 but emphasize total field amplitude rather than directional components, further supporting the strong subwavelength confinement required for broadband absorption and biochemical detection.

Fig 12 illustrates the real and imaginary components of the magnetic field (Hz). Relative to electric-field intensities, the magnetic-field range (216–245 dB μA/m) is narrower, but the spatial evolution remains consistent with plasmonically induced magnetic dipole resonances.

The real component (Fig 12(a)) shows predominantly blue regions with isolated green–yellow patches along the resonator edges, indicating initiation of circulating displacement currents. The imaginary component (Fig 12(b)), however, exhibits broader zones of enhanced magnetic activity, particularly near the inner ring boundaries and bridging sections. Although magnetic-field intensities do not reach values as high as the electric fields, the localized yellow regions represent the primary magnetic hot spots responsible for strong magnetic dipolar coupling across the 0.1–30 THz band.

Fig 13 provides the magnitude of the magnetic field (|H|) using the same 216–245 dB μA/m scale. Both real (Fig 13(a)) and imaginary (Fig 13(b)) components reveal low-intensity blue regions in outer areas and progressively stronger confinement toward the metal edges and the split-ring gaps. Yellow–red regions correspond to the highest |H|-field concentrations, demonstrating where magnetic dipole resonances are maximized.

The imaginary component again displays more prominent red and orange hot spots, highlighting areas of strong reactive magnetic energy and efficient light trapping. These resonant magnetic features are fundamental to perfect absorption, as they create conditions where electric and magnetic responses are simultaneously optimized.

**Fig 14:**
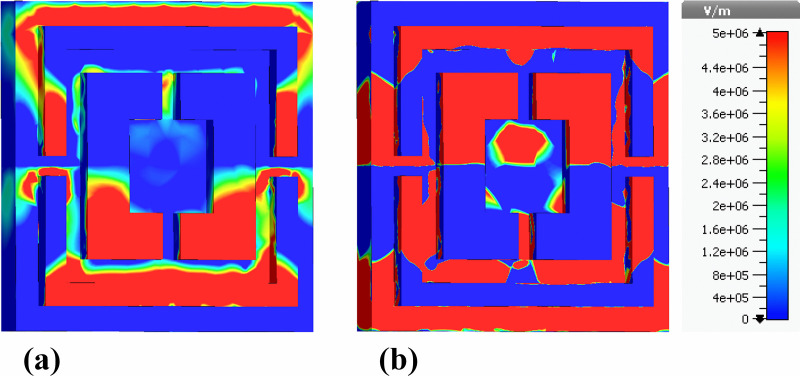
Back-plane representation of the nickel ground layer showing nanophotonic electric-field distributions: (a) real part of the out-of-plane electric field component (Ez) and (b) imaginary part of the out-of-plane electric field component (Ez).

The images provide detailed visualizations of the electric field (Ez-field) distributions on the rear side of a nano-photonic biosensor, which is engineered based on a perfect absorber design. This biosensor is optimized for ultra-wideband operation within the 0.1 THz to 30 THz frequency range, enabling high-sensitivity detection of biomolecular interactions. The device incorporates a nickel ground layer that plays a crucial role in its electromagnetic properties. Fig 14(a) demonstrates the real components of the Ez-field, while Fig 14(b) highlights the imaginary components. Both figures are represented using color gradients that depict varying intensities of the electric field, with a scale ranging from blue (indicating lower intensities) to red (indicating higher intensities), corresponding to electric field magnitudes between 0 and 5e + 6 V/m.

The field distributions in both components of Fig 1 are concentrated around the geometric features of the nanophotonic ground plane. This suggests strong confinement and manipulation of electromagnetic waves by the nano-photonic structures. The intensity variations across the ground surface signify regions where electromagnetic energy is most efficiently captured and absorbed, enhancing the overall performance of the biosensor. Such field localization is vital for improving the sensor’s sensitivity, especially in the terahertz regime, where it can detect minor changes in the dielectric properties of biological materials. The distinction between the real and imaginary field components reveals different resonant behaviors, with the real component being more associated with energy storage and the imaginary component providing insight into the loss mechanisms and absorption efficiency.

**Fig 15:**
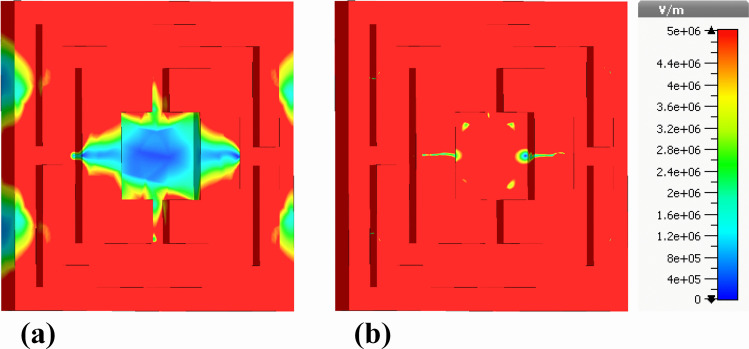
Rear-side color-mapped profiles of the electric-field magnitude within the nickel ground plane, illustrating (a) real component of |E| and (b) imaginary component of |E|.

Similarly, Fig 15 presents the electric field distributions of the same nano-photonic biosensor, but here the emphasis is placed on the absolute value of the electric field (|E|-Field). This figure portrays the overall field intensity at the rear side, offering a comprehensive view that combines contributions from all directional components. The nickel ground layer is again illustrated, facilitating effective absorption of electromagnetic waves. The absolute electric field intensities are shown using a similar color gradient, with a range between 0 and 5e + 6 V/m, enabling a clear representation of the electric field magnitude at different points on the ground layer.

In contrast to Fig 14, which isolates the z-axis components of the electric field, Fig 15 provides a more holistic perspective on the total field strength across the entire sensor. This comprehensive mapping is essential for evaluating the sensor’s electromagnetic performance since it reveals how well the nanophotonic design captures, concentrates, and dissipates electromagnetic energy. By analyzing both the real and imaginary field components, in conjunction with the absolute field values, researchers can better understand the resonance conditions and the sensor’s overall effectiveness in detecting biomolecular interactions. This combination of field distributions provides critical insights into the sensor’s impedance matching, resonant behaviors, and absorption efficiency, all of which are foundational in optimizing the biosensor for non-invasive diagnostics within the terahertz frequency range.

**Fig 16:**
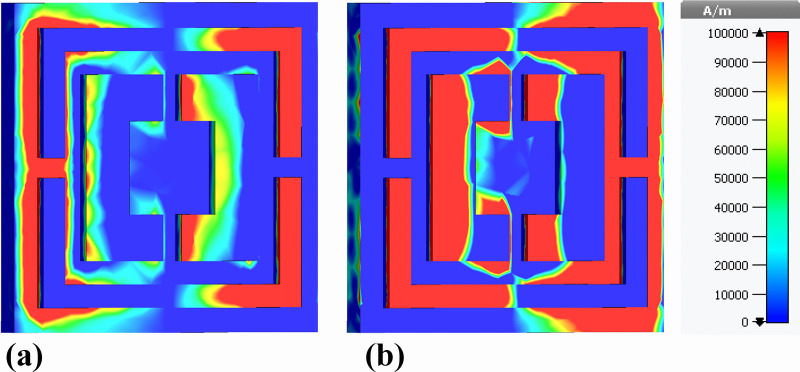
Back-view visualization of magnetic-field behavior at the nickel ground layer: (a) real part of the out-of-plane magnetic field (Hz) and (b) imaginary part of the out-of-plane magnetic field (Hz).

Fig 16(a) presents the real component of the |H|-field distribution within a nanophotonic biosensor structure. The localized regions of high magnetic field intensity (1e + 5 A/m), as visualized by the color gradient, indicate the presence of resonant modes within the nanostructure. These resonances arise from the interaction of the incident terahertz radiation with the engineered nanostructures, leading to efficient coupling and confinement of electromagnetic energy. The observed field distribution patterns suggest that the nanophotonic design is optimized for selective absorption and detection of terahertz waves.

Fig 16(b) illustrates the imaginary component of the |H|-field distribution. This component reveals the phase shifts and wave propagation characteristics within the nanostructure. The spatial phase variation indicates areas where the electromagnetic fields are being dynamically absorbed and redistributed. The interplay between the real and imaginary components of the magnetic field is crucial for understanding the energy dissipation mechanisms within the material, as the phase differences contribute to the overall absorption efficiency and impedance matching.

**Fig 17:**
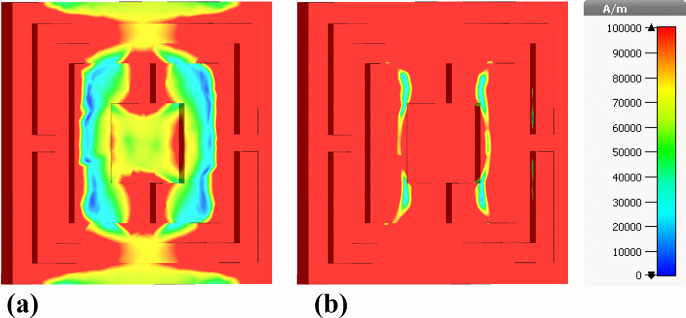
Spatial distributions of the magnetic-field magnitude across the nickel ground layer obtained from rear-side simulations, showing (a) real part of |H| and (b) imaginary part of |H|.

Fig 17(a) presents the real component of the |H|-field distribution for a slightly modified nanophotonic structure. While the overall trend remains similar to Fig 16(a), the field distribution patterns are more refined and localized to specific regions of the structure. This suggests that the geometric modifications have introduced new resonant modes or altered the existing ones, resulting in a more focused interaction with the incident terahertz radiation.

Fig 17(b) shows the imaginary component of the |H|-field for the modified nanophotonic structure. The phase variation is more uniform compared to Fig 16(b), indicating a more balanced interaction between the electromagnetic waves and the material. This suggests that the modifications have improved the impedance matching between the biosensor and free space, leading to enhanced absorption efficiency.

While all four Figures (Fig.14, Fig 15, Fig 16 and Fig 17) demonstrate the presence of resonant magnetic field localization and surface plasmonic effects, there are notable differences in the field distribution patterns and phase behaviors. Figs 16 and 17 exhibit more localized and refined field distributions, suggesting a more focused interaction with specific resonant modes. This may be attributed to the variations in the nanophotonic design. In contrast, Figs 14 and 15 show a more uniform field distribution, indicating a broader interaction with the incident radiation. Both Figs 16 and 17 provide valuable insights into the localized resonances and phase dynamics within the nanophotonic biosensor. These observations contribute to a deeper understanding of the underlying mechanisms governing the sensor’s performance and offer guidance for future optimization efforts.

### 5.2. Analysis of surface current characteristics and angular response behavior

**Fig 18:**
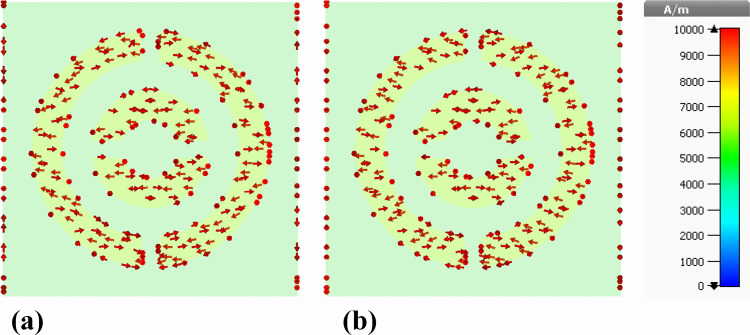
Front-plane depiction of surface current density distributions on the silver resonator array of the proposed nanophotonic absorber, illustrating (a) real and (b) imaginary components.

The efficacy of the designed nanophotonic absorber can be assessed through the analysis of current distribution along its top and bottom surfaces. To examine this, we plotted the current distribution diagrams of the front surface containing the silver resonator and the rear surface consisting of the nickel ground layer. Fig 18 illustrates the current distribution diagrams of the silver resonator surface for both the real and imaginary components of the electric field. It was observed that the current distribution follows the same pattern for both components and is uniformly distributed over the silver resonator surface.

**Fig 19:**
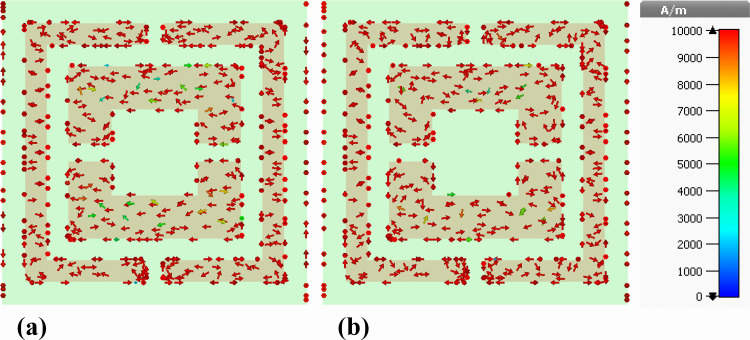
Rear-side representation of surface current density profiles induced on the nickel ground layer of the proposed nanophotonic structure, showing (a) real and (b) imaginary components.

Fig 19 demonstrates the current distribution diagrams of the rear surface of the proposed structure containing the nickel ground layer. For both real and imaginary components of the electric field, the intensity of the current is higher in the outer ring compared to the inner ring. Regarding the direction of current flow, on both the front and rear surfaces, the current flows from right to left, creating a parallel direction. This leads to the generation of electric resonance along the front and rear surfaces, enhancing the light interaction with the proposed structure and resulting in improved absorption.

**Fig 20:**
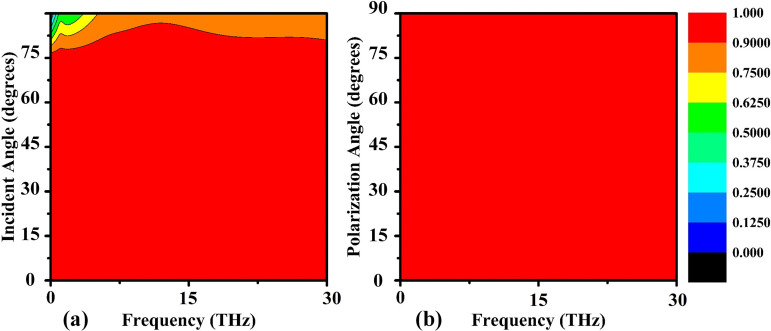
Parametric assessment of angular dependence on absorption characteristics: (a) effect of varying the electromagnetic wave incidence angle and (b) effect of polarization angle rotation on absorption performance.

The aforementioned analysis discussed the absorption property of incident light at a normal angle of incidence. However, in practical applications, light often incidents on the device at an oblique angle. Therefore, it is essential to study the absorption characteristics over a wide range of incident angles. Fig 20(a) depicts the variation in the absorption property of the designed nanophotonic absorber as the incident angle is changed from 0 to 90 degrees. It is observed that the proposed structure acts as a nearly perfect absorber with an absorption efficiency of more than 90% up to an incident angle of 76 degrees. This variation in absorption characteristics is due to the retention of the magnetic field during changes in the incident angle. Furthermore, the absorption efficiency decreases to 75% for incident angles greater than 76 degrees.

Fig 20(b) shows the analysis of the absorption performance of the proposed structure with respect to different polarization angles ranging from 0 to 90 degrees. It is evident that the structure behaves as a perfect absorber with an absorption efficiency of more than 90% for a broad range of polarization angles.

Based on the aforementioned analysis, it can be concluded that the proposed nanophotonic structure can act as a nearly perfect stable absorber even at a wide range of oblique incident angles and polarization angles.

## 6. Benchmarking the nanophotonic perfect absorber

The advancement of nanophotonic absorbers has accelerated in recent years, particularly in applications requiring compact geometry, wide spectral coverage, and high absorption efficiency. In this context, benchmarking the proposed nanophotonic perfect absorber against established designs is essential for objectively assessing its technical relevance and contribution to the field. This section provides a data-driven comparison emphasizing operating bandwidth, absorption uniformity, device compactness, and suitability for biomedical and environmental sensing.

### 6.1. Performance characteristics relevant to biomedical sensing

The proposed nanophotonic absorber exhibits broadband operation extending from 100 GHz to 30 THz, representing a substantially wider range than most reported designs, which typically achieve up to 15.9 THz (Table 2). This broad spectral response is accompanied by near-uniform absorption exceeding 99.92% across the entire band.

**Table 2:** Comparative Analysis with Relevant Work.

Ref.	Materials	Unit cell Dimension (nm)	Operation Range (THz)	Bandwidth (THz)	Polarization Insensitivity	Angular Stability	Absorption	Size Scale	Nanoscale Sensing Capability
[79]	Vo_2,_Polyimide,Au	24,000 × 24,000 × 10,000	2.54–5.54	3	yes	90% absorption for θ ≤ 80	>90%	Micron	No
[80]	Vo_2_, SiO_2,_Au	35,000 × 35,000 × 13,850	8.5–11	2.5	yes	–	> 99%	Micron	No
[81]	Vo_2,_Quartz,Au	28,000 × 28,000 × 8,200	3.05–8.14	5.09	yes	90% absorption for θ ≤ 60	>90%	Micron	No
[82]	Vo_2_ SiO_2,_Au	30,000 × 30,000 × 9,200	2.34–5.64	3.30	yes	90% absorption for θ ≤ 80	>90%	Micron	No
[77]	Graphene,polyimide,Au	33,000 × 33,000 × 36,000	0.80–1.87	1.07	yes	80% absorption for θ ≤ 60	> 99%	Micron	No
[83]	Vo_2_, Topas_,_Au	30,000 × 30,000 × 20,000	1.63–12.39	10.76	yes	90% absorption for θ ≤ 80	>90%	Micron	No
[84]	ITO, PET	55,000 × 55,000 × 15,000	1.75–5	3.25	yes	90% absorption for θ ≤ 60	>90%	Micron	No
[85]	Graphene, SiO_2_,Au	25,000 × 25,000 × 25,000	7–9.5	2.5	yes	90% absorption for θ ≤ 70	>90%	Micron	No
[86]	Vo_2_,SiO_2_, Au	24,000 × 24,000 × 7,000	3.01–7.27	4.26	yes	90% absorption for θ ≤ 80	>90%	Micron	No
[87]	Vo_2,_Quartz,Au	75,000 × 75,000 × 11,000	2.7–5.7	3	yes	90% absorption for θ ≤ 80	>90%	Micron	No
[78]	ECCOSORB AN-72, SiO_2_Ni	40,000 × 40,000 × 870,000	0.1–16	15.9	yes	90% absorption for θ ≤ 80	>95%	Micron	No
This work	Ag, SiO_2,_Ni	100 × 100 × 26	0.1–30	30	yes	90% absorption for θ ≤ 78	>99.92%	Nano	Yes

The structure’s polarization-insensitive behavior further enhances its reliability, as it maintains consistent performance regardless of incident field orientation. This feature is particularly relevant for biosensing scenarios where the polarization state may not be controlled.

The geometric simplicity and extremely compact design—comprising a 100 × 100 nm² footprint and a total thickness of 26 nm using Ag, SiO₂, and Ni—contribute to ease of fabrication and integration into nanoscale diagnostic platforms. These attributes collectively support the absorber’s ability to detect minute refractive index variations, which is essential for label-free biomedical sensing and characterization of biological tissues.

### 6.2. Application scope and alignment with current research directions

The absorber’s broadband coverage from the THz to the infrared region positions it well for applications where wide spectral information is advantageous, including tissue characterization, chemical detection, and environmental monitoring. Its sensitivity to refractive index changes enables potential use in early-stage disease detection, where small dielectric contrasts are diagnostically meaningful.

Beyond biomedical applications, the device’s spectral range and angular stability support its applicability in chemical vapor sensing, pollutant monitoring, and THz/IR imaging. These areas have seen increasing research interest due to the demand for compact, high-resolution detection tools.

The absorber’s operational and structural features therefore align with established research directions in nanophotonics, metamaterial sensing, and multi-band environmental diagnostics, offering a technically grounded pathway for broader deployment.

### 6.3. Comparative assessment and scientific benchmarking

To evaluate the contribution of the proposed design within the broader literature, a comparative analysis was conducted using Tables 3 and 4, which summarize performance metrics, material systems, and architectural choices of leading absorbers. When benchmarked against these designs, the proposed device demonstrates:

**Table 3:** Comparative Performance Metrics with Existing Absorbers.

Feature	Proposed Sensor	State-of-the-Art Absorbers	Novelty/Advantage
**Operating Frequency Range**	100 GHz – 30 THz	Up to ~15.9 THz	Nearly 2 × broader bandwidth
**Absorption Efficiency**	>99.92%	~90%–99%	Near-perfect absorption
**Structural Thickness**	26 nm total	Typically >7000 nm	Ultra-thin design
**Device Footprint**	100 × 100 nm²	Micro-scale (μm) structures	Nanoscale resolution
**Material Composition**	Ag, Ni, SiO₂	Varies (often Au, Cu, polymers, Vo_2_)	Cost-effective, CMOS-compatible
**Polarization Sensitivity**	Insensitive	Often sensitive	Reliable under varying polarization
**Refractive Index Sensitivity**	Ultra-sensitive (early-stage cancer)	Moderate	Detects subtle biological changes
**Application Suitability**	Biomedical, security, environmental	Limited to biomedical or single-purpose use	Highly versatile

**Table 4:** Key Innovation Aspects of the Proposed Nano-Photonic Absorber.

Innovation Parameter	Proposed Sensor Attribute	Significance
**Detection Scale**	Nanoscale (100 × 100 nm²)	Enables ultra-sensitive detection, surpasses microscale systems
**Operating Bandwidth**	Ultra-wideband (100 GHz – 30 THz)	Covers THz to IR, supporting diverse sensing applications
**Absorptivity**	>99.92% (Broadband near-perfect)	Ideal for biomedical and stealth applications
**Structural Compactness**	Total thickness: 26 nm	Facilitates integration into miniaturized devices
**Material Optimization**	Ni/Ag resonators + SiO₂ substrate	Enhances electrical/magnetic response while reducing cost
**Angular & Polarization Stability**	Stable across angles and polarization states	Reliable real-world performance
**Application Domains**	Cancer diagnostics, pollutant detection, security	Supports next-gen biomedical and environmental technologies
**Benchmark Superiority**	Outperforms in size, bandwidth, efficiency	Sets a new performance benchmark in nanophotonic sensing

1) A significantly wider bandwidth (100 GHz–30 THz vs. ≤ 15.9 THz),
2) Near-perfect and uniform absorption (>99.92%) over the entire range,
3) Substantial miniaturization, with structural dimensions at the nanoscale rather than the microscale,
4) Polarization insensitivity and angular stability, which are not commonly achieved simultaneously in comparable absorbers.

These improvements stem from the engineered interaction between the Ag and Ni resonators and the SiO₂ dielectric layer, which enhances impedance matching and field confinement across the spectrum.

The device’s capability to detect small refractive index variations arises from these localized field enhancements and the ultra-thin geometry, which amplifies the interaction volume between the sensing medium and the electromagnetic field.

Taken together, the benchmarking results indicate that the absorber provides measurable improvements in spectral coverage, structural compactness, and functional versatility relative to established designs. Rather than presenting an incremental modification, the device introduces a combination of nanoscale footprint, wideband performance, and uniform absorption that distinguishes it within current nanophotonic absorber research.

## 7. Future perspectives

The proposed nanophotonic perfect absorber demonstrates key characteristics—ultra-broadband operation, high absorption efficiency, nanoscale thickness, and polarization-insensitive behavior—that suggest its potential suitability for several practical sensing applications. While the present work focuses on numerical validation, the underlying physical mechanisms indicate that the device may support future developments in biomedical and chemical detection where sensitivity to refractive-index variations is critical.

In biomedical diagnostics, the strong electromagnetic field confinement exhibited by the structure could be advantageous for identifying subtle dielectric differences in biological samples. Although further experimental investigation is required, these properties align with the needs of early-stage tissue characterization and label-free detection approaches commonly used in terahertz and infrared biosensing.

Beyond biomedical applications, the absorber’s broadband response may be relevant for chemical and environmental monitoring, particularly where analytes exhibit spectral signatures in the terahertz-to-infrared range. The compact footprint of the device also suggests potential compatibility with integrated photonic platforms, enabling the possibility of miniaturized sensing modules for analytical or point-of-care systems after appropriate fabrication and calibration steps.

Advances in nanophotonic design, fabrication tolerances, and hybrid-material engineering may further refine the absorber’s performance and stability. Future studies may also explore coupling the structure with data-driven analysis tools to support automated interpretation of spectral shifts, especially in complex biological or environmental settings. Ultimately, while additional experimental validation is needed to assess practical performance, the numerical results presented here provide a foundation that may inform subsequent research toward compact, broadband nanophotonic sensors for biomedical and chemical detection tasks.

## 8. Conclusions

This research focused on developing an innovative, compact THz-range sensor for biomedical applications. The design prerequisites included ensuring compact size, wide operating bandwidth, and geometrical simplicity. The proposed sensor consists of two meticulously dimensioned resonators implemented using silver (Ag) and nickel (Ni), allocated on a silicon dioxide (SiO2) dielectric substrate. The overall dimensions of the device are 100 × 100 nm². At the same time, the total thickness of the Ag, SiO2, and Ni layers is merely 26 nm. The paper details the sensor’s development process and discusses the evolution of the system’s architecture. Extensive numerical studies have been carried out to demonstrate the sensor’s excellent performance, analyzed through surface current, electric and magnetic field distributions, scattering parameters, and refractive index. One of the essential characteristics of the device is near-perfect absorptivity (>99.9%) across the broad operating range up to 30 THz. Comprehensive comparative studies conducted for several state-of-the-art benchmark structures reveal the sensor’s superior performance regarding operating range, compact size, absorption efficiency, angular stability, and the ability to detect subtle changes in tissue refractive index, making it suitable for early-stage cancer diagnostics and other biomedical applications. It is also well-suited for detection of chemical compounds and security screening.

## 9. Feasibility and experimental implementation of the nanophotonic absorber

The structural parameters and material selection for the proposed nanophotonic absorber, while derived from full-wave numerical simulations, were deliberately chosen to ensure practical fabrication feasibility. The multilayer geometry, comprising Ag and Ni nanoscale resonators separated by a thin SiO2 dielectric, fits within the capabilities of contemporary nanofabrication technologies. Specifically, the compact ~100 × 100 nm² footprint and ~26 nm total thickness are achievable using established high-resolution techniques. Electron-beam lithography (EBL) can define the nanoscale Ag patterns with sub-10-nm precision, while Atomic Layer Deposition (ALD) or e-beam evaporation/magnetron sputtering can realize the precise thickness control required for the SiO2, Ag, and Ni films. Despite this compatibility, successful translation from simulation to prototype necessitates stringent control over nanoscale manufacturing tolerances and the use of high-precision metrology, such as spectroscopic ellipsometry and atomic-force microscopy, to validate structural fidelity, as minor thickness or roughness deviations can induce resonance shifts.

The ultra-broadband spectral characterization of the absorber (0.1–30 THz) requires a segmented measurement strategy due to the absence of a single system spanning this range. Experimental validation of the absorption response involves combining Terahertz Time-Domain Spectroscopy (THz-TDS) for the low-frequency range (0.1–10 THz) with Fourier-Transform Infrared Spectroscopy (FTIR) to cover the mid-infrared performance up to ~30 THz. Furthermore, Near-field Scanning-Optical-Microscopy (NSOM) is essential for obtaining spatially resolved field distributions to correlate with simulated near-field maps. Following the fabrication and characterization of the optical constants for the constituent materials, the experimentally derived reflection and absorption spectra will be critically compared against the modeled results. This comparison will quantify the influence of inherent material losses, environmental factors, and fabrication deviations not captured by idealized theoretical models. The technically grounded roadmap (Table 5)—from EBL patterning of the multilayer stack to segmented spectral measurement—provides a viable pathway for the experimental validation and subsequent integration of this ultra-broadband absorber into functional platforms for biosensing and other applications.

**Table 5:** Technically Grounded Roadmap for Absorber Validation.

Stage	Objective	Key Techniques	Description/Goal
**i. Nanofabrication**	Realize the Ag–SiO2–Ni multilayer structure.	Electron-Beam Lithography (EBL)	Pattern nanoscale Ag resonators with sub-10-nm accuracy.
		Atomic Layer Deposition (ALD)	Achieve conformal, precise thickness control for the SiO2 dielectric.
		E-beam Evaporation/Sputtering	Deposit Ag and Ni films with precise film thickness control (~26 nm total thickness).
**ii. Structural Metrology**	Validate structural fidelity and dimensional accuracy.	Atomic-Force Microscopy (AFM)	Verify device footprint (~100 × 100 nm²) and surface roughness.
		Spectroscopic Ellipsometry	Verify film thicknesses and dispersive optical constants of Ag, Ni, and SiO2.
**iii. Spectral Characterization**	Validate the ultra-broadband absorption (0.1–30 THz).	THz Time-Domain Spectroscopy (THz-TDS)	Measure reflection/absorption in the 0.1–10 THz range.
		Fourier-Transform Infrared Spectroscopy (FTIR)	Measure reflection/absorption in the mid-infrared up to ~30THz.
		Near-Field Scanning Optical Microscopy (NSOM)	Obtain spatially resolved near-field distributions for comparison with simulations.
**iv. Validation & Integration**	Compare experimental results to simulations and plan for practical application.	Data Analysis & Comparison	Assess influence of fabrication tolerances and intrinsic material losses on resonance shift.
		Device Integration Planning	Outline future developments (e.g., microfluidic channels, chip-scale emitters) for biosensing platform.
